# Identification of a fluorometabolite from *Streptomyces* sp. MA37: (2*R*3*S*4*S*)-5-fluoro-2,3,4-trihydroxypentanoic acid†
†Electronic supplementary information (ESI) available: The *fdr* gene cluster was deposited in EMBL Nucleotide Sequence Database under the accession no: LN612605. See DOI: 10.1039/c4sc03540b


**DOI:** 10.1039/c4sc03540b

**Published:** 2014-12-03

**Authors:** Long Ma, Axel Bartholome, Ming Him Tong, Zhiwei Qin, Yi Yu, Thomas Shepherd, Kwaku Kyeremeh, Hai Deng, David O'Hagan

**Affiliations:** a EaStChem School of Chemistry , University of St Andrews , North Haugh , St Andrews KY169ST , UK . Email: do1@st-and.ac.uk; b Marine Biodiscovery Centre , Department of Chemistry , University of Aberdeen , Meston Walk , Aberdeen AB24 3UE , UK . Email: h.deng@abdn.ac.uk; c Key Laboratory of Combinatory Biosynthesis and Drug Discovery (Ministry of Education) , School of Pharmaceutical Sciences , Wuhan University , 185 East Lake Road , Wuhan 430071 , P. R. China; d The James Hutton Institute , Invergowrie , Dundee , DD2 5DA , UK; e Department of Chemistry , University of Ghana , FGO Torto Building , Legon , Ghana

## Abstract

(2*R*3*S*4*S*)-5-Fluoro-2,3,4-trihydroxypentanoic acid (5-FHPA) has been discovered as a new fluorometabolite in the soil bacterium *Streptomyces* sp. MA37. Exogenous addition of 5-fluoro-5-deoxy-d-ribose (5-FDR) into the cell free extract of MA37 demonstrated that 5-FDR was an intermediate to a range of unidentified fluorometabolites, distinct from fluoroacetate (FAc) and 4-fluorothreonine (4-FT). Bioinformatics analysis allowed identification of a gene cluster (*fdr*), encoding a pathway to the biosynthesis of 5-FHPA. Over-expression and *in vitro* assay of FdrC indicated that FdrC is a NAD^+^ dependent dehydrogenase responsible for oxidation of 5-FDR into 5-fluoro-5-deoxy-lactone, followed by hydrolysis to 5-FHPA. The identity of 5-FHPA in the fermentation broth was confirmed by synthesis of a reference compound and then co-correlation by ^19^F-NMR and GC-MS analysis. The occurrence of 5-FHPA proves the existence of a new fluorometabolite pathway.

## Abstract 2

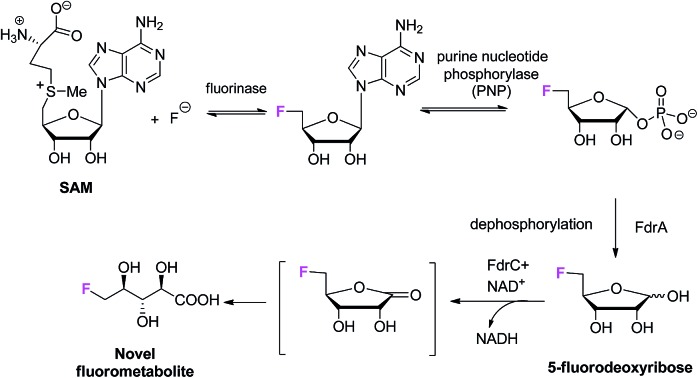
(2*R*3*S*4*S*)-5-Fluoro-2,3,4-trihydroxypentanoic acid (5-FHPA) has been discovered as a new fluorometabolite in the soil bacterium *Streptomyces* sp. MA37.

## Introduction

The introduction of fluorine into organic molecules can substantially modulate their physicochemical properties.^1^ About 30% of current drugs, including many top sellers, contain at least one fluorine atom.^2^ In contrast to man-made molecules, fluorinated natural products are extremely rare.^3^

**Scheme 1:**
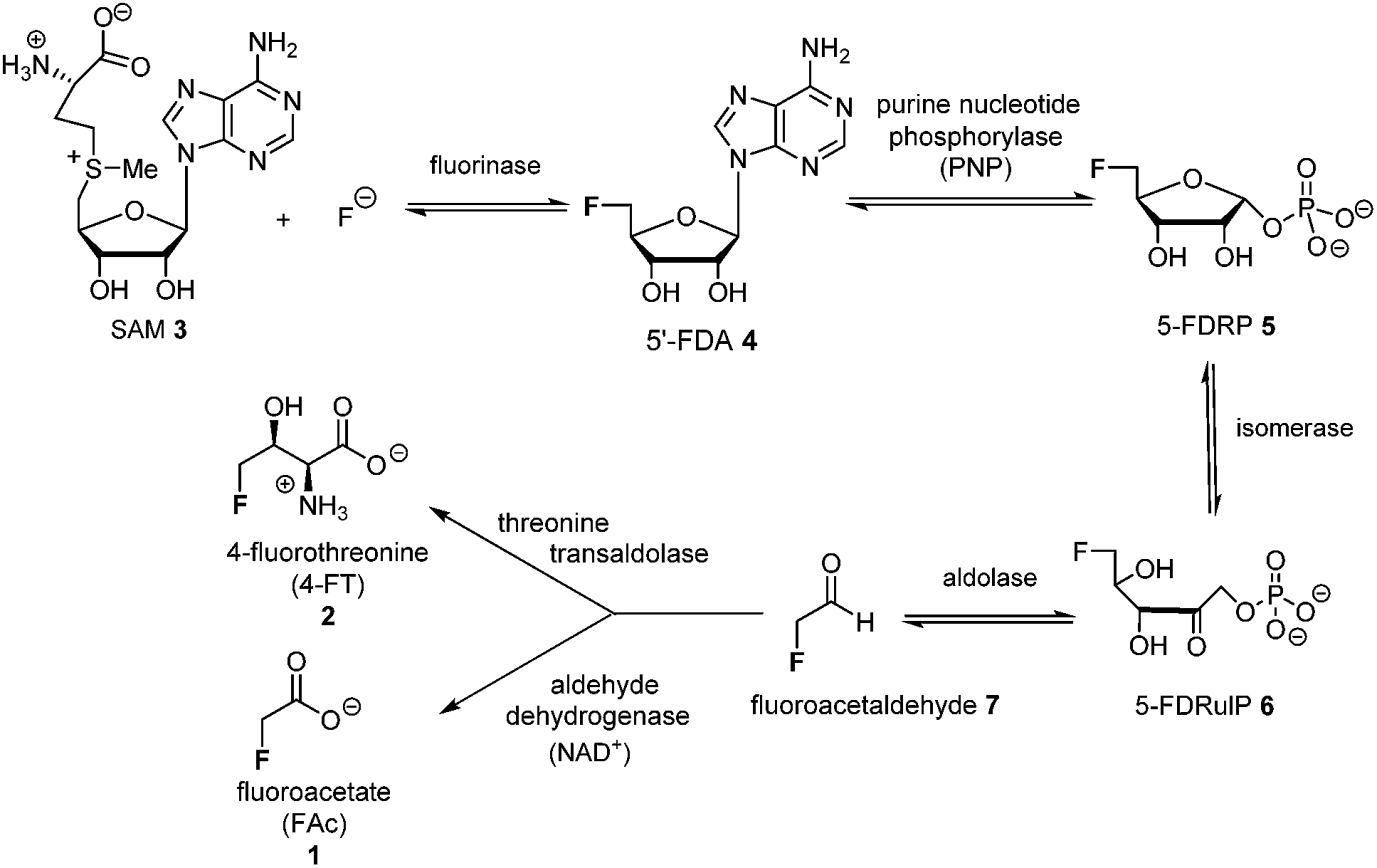
Biosynthetic pathway to FAc **1** and 4-FT **2** in actinomycete bacteria.

Fluoroacetate (FAc) **1** is the most ubiquitous fluorometabolite found as a toxic self-defence agent in many tropical and sub-tropical plants.^4^ In 1986, the actinomycete soil bacterium *Streptomyces cattleya* was found to secrete FAc **1** and 4-fluorothreonine (4-FT) **2** as part of its metabolic profile when grown in the presence of inorganic fluoride.^5^ Over the last decade, intermediates on the fluorometabolite pathway in *S. cattleya* have been uncovered and are shown in Scheme 1.^6^ The formation of the C–F bond is catalysed by the fluorinase, which mediates the biotransformation of *S*-adenesyl-l-methionine (SAM) **3** and inorganic fluoride into 5′-fluoro-5-deoxy-adenosine (5′-FDA) **4**.^7^ 5′-FDA is then phosphorylated generating 5-fluoro-5-deoxy-ribose phosphate (5-FDRP) **5**,^8^ followed by ring opening to generate 5-fluoro-5-deoxy-ribulose-phosphate (5-FDRulP) **6**.^9^ An aldolase catalyses a retro-aldol reaction to generate fluoroacetaldehyde **7**, the last common intermediate on the pathway.^10^ Fluoroacetaldehyde **7** is either oxidised to FAc **1**,^11^ or biotransformed into 4-FT **2** catalysed by a PLP-dependent transaldolase.^12^ The fluorinase gene remained a sole representative in the genome databases for a decade, however between 2012–2014, three fluorinase genes appeared in the genomes of sequenced microorganisms (*Streptomyces* sp. MA37, *Actinoplanes* sp. N902-109,^13^*Norcardia brasiliensis*^13^,^14^). PCR amplification of these genes from genomic DNA, or the expression of synthetic genes in *E. coli* demonstrated that these were all functional fluorinases. Most recently, the marine actinomycete *Streptomyces xinghaiensis* (NRRL B-24674) was also shown to have a functional fluorinase.^15^*S. xinghaiensis* was found to produce FAc **1** only (no 4-FT **2**) and FAc **1** production is sea-salt dependent.^15^*S. xinghaiensis* is the first fluorometabolite producer from a marine microorganism. Most importantly, all fluorinase genes described after the disclosure of that obtained from *S. cattleya* have greater than 80% sequence identity to the original fluorinase. In culture, *N. brasiliensis* was unable to produce a trace of fluorometabolite under laboratory culture conditions.^13^,^14^

*Streptomyces* sp. MA37, an isolate from a Ghanaian soil sample, produces FAc **1** and 4-FT **2** in culture.^13^ Unlike *S. cattleya* and *S. xinghaiensis*, *Streptomyces* sp. MA37 also produced a range of unidentified fluorometabolites in addition to FAc **1** and 4-FT **2**, as determined by ^19^F-NMR of a supernatant extract.^13^ The identity of these novel fluorometabolites is of interest given that this class of natural products is so rare.

Here we report that 5-FDRP **5** is a branch point metabolite between two pathways. Exogenous addition of 5-fluoro-5-deoxyribose (5-FDR) **8** to cell free extracts was found to support biosynthesis predominantly of the unidentified fluorometabolites and could not significantly support FAc **1** and 4-FT **2** biosynthesis. Genomic-driven analysis allowed identification of the *fdr* gene cluster, encoding elements of the new pathway. *In vitro* assay of over-expressed FdrC demonstrated that the protein is a NAD^+^ dependent dehydrogenase that oxidizes 5-FDR **8** to 5-fluoro-5-deoxy-d-ribono-γ-lactone (5-FRL) **9**, followed by lactone hydrolysis to (2*R*3*S*4*S*)-5-fluoro-2,3,4-trihydroxypentanoic acid (5-FHPA) **10**. This sequence from 5-FDRP forms the first steps of a new fluorometabolite pathway.

## Results and discussion

**Scheme 2:**
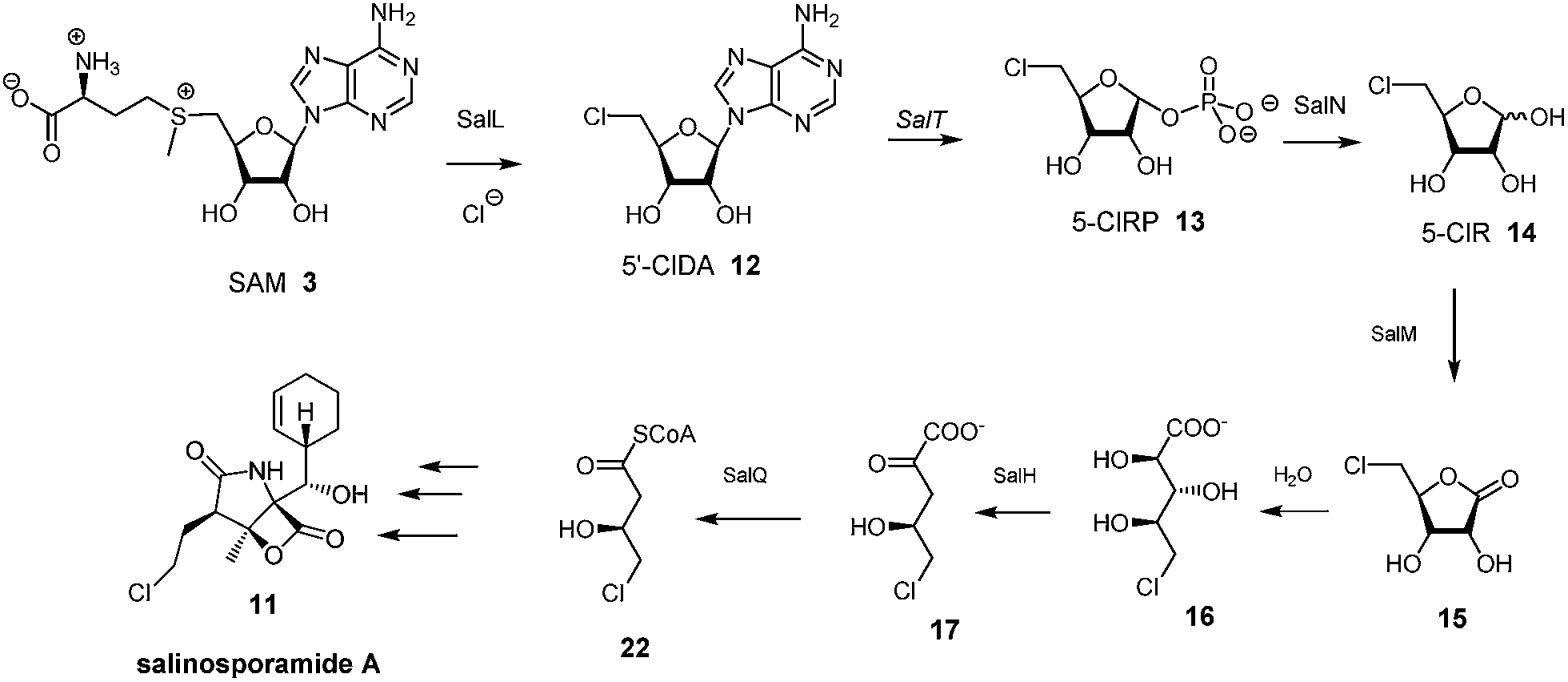
Early steps in salinosporamide A **11** biosynthesis.^17^

5-FDRP **5** is known to be an intermediate in the biosynthesis of FAc **1** and 4-FT **2** in *Streptomyces cattleya*.^8^ In that study it was established that the de-phosphorylated free ribose 5-FDR **8** was unable to recover fluorometabolite biosynthesis in cell free extracts. It was unable to support fluorometabolite biosynthesis, and it was also unable to become phosphorylated to re-generate **5** and channel back into the fluorometabolite pathway to generate FAc **1** or 4-FT **2**.^8^,^16^ Interestingly in the marine microorganism *Salinispora tropica*, a closely related pathway operates as illustrated in Scheme 2.^17^ This bacterium produces salinosporamide A **11** a chlorinated metabolite which has received attention as an anti-cancer therapeutic.^18^ Investigations into salinosporamide-A **11** biosynthesis have shown that the chlorine is introduced by a SAM-dependent chlorinase to generate 5′-ClDA **12**, an enzyme closely analogous to the fluorinase of *S. cattleya*.^19^ The two pathways also share the second step. In *S. cattleya*, depurination of 5′-FDA generates 5-FDRP **5** and analogously in *S. tropica* 5′-ClRP **13** is generated. The two pathways appear to diverge at this point.^17^ For salinosporamide-A **11**, SalN catalyses dephosphorylation of 5-ClRP **13** to generate the free sugar 5-chloro-5-deoxy-d-ribose (5-ClR) **14** as a key intermediate on the biosynthetic pathway to salinosporamide A **11**. The free sugar does not appear to be relevant in *S. cattleya*. Interestingly, 5-FDR **8** was also found to support this biotransformation to form the analogous fluorinated derivative of salinosporamide A, demonstrating that the salinosporamide-A pathway can channel fluoromethyl as well as chloromethyl intermediates.^20^

**Fig. 1:**
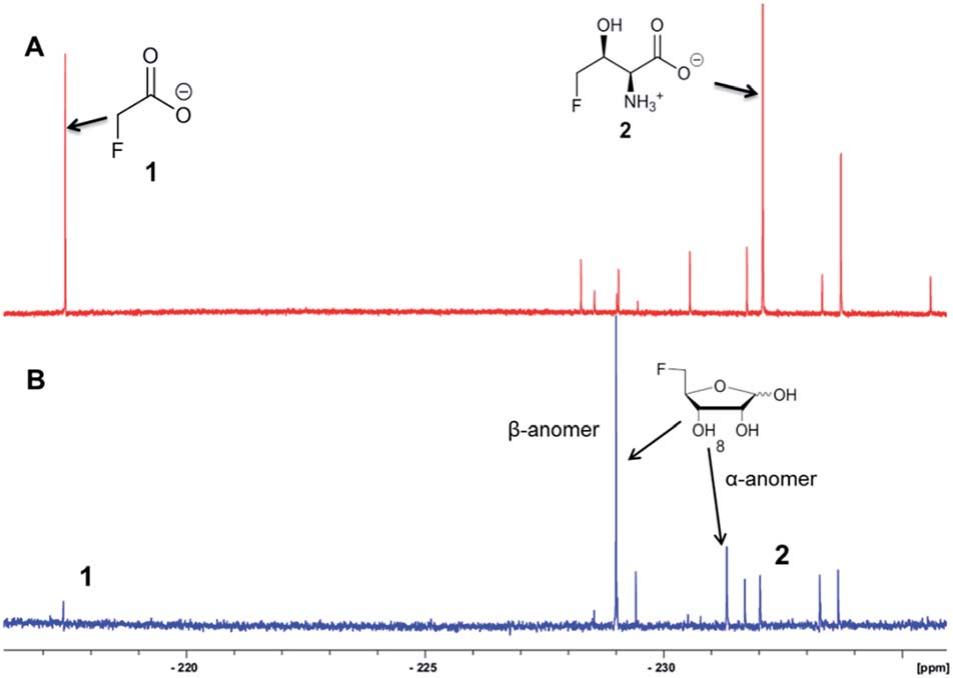
^19^F NMR spectroscopic analyses of fluorometabolites: (A) fluoroacetate, 4-fluorothreonine and seven unidentified fluorometabolites in the supernatant of cultures from *Streptomyces* sp. MA37; (B) fluoroacetate, 4-fluorothreonine, and four unidentified fluorometabolites in the cell free extract of MA37 strain incubated with 5-FDR **8**. The signals of excess exogenously added 5-FDR **8** are highlighted.

In this context it appeared appropriate to explore a role for 5-FDR **8** in *Streptomyces* sp. MA37 metabolism to establish if any of the additional unknown metabolites are derived from the free sugar. To this end, 5-FDR **8** was prepared by synthesis^8^ and was incubated with a cell-free extract (CFE) of *Streptomyces* sp. MA37. Products were monitored by ^19^F-NMR. The usual dominance of FAc **1** and 4-FT **2**, found in full whole cell incubations with added fluoride ion was no longer the observed profile. Instead these signals were substantially diminished and some of the minor unknowns now dominated the ^19^F-NMR spectrum (Fig. 1). These unknown signals were not observed in control experiments using boiled CFE incubated with 5-FDR **8** or the CFE alone. Therefore 5-FDR **8** appears to be an intermediate to some of the fluorometabolites in *Streptomyces* sp. MA37, and does not appear to support FAc **1** or 4-FT **2** biosynthesis in a primary manner.

**Fig. 2:**
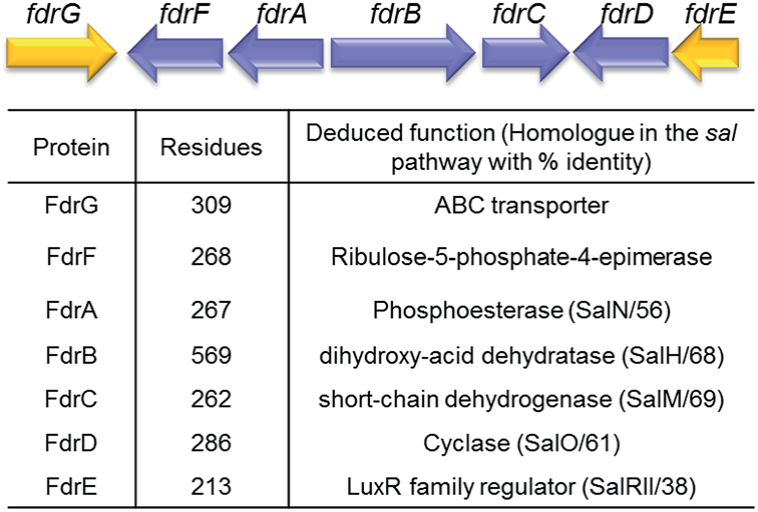
Organisation and proposed functions of the encoded proteins of the identified fluorometabolite gene cluster (8.55 kb; accession no. LN612605) predicted to direct the biosynthesis of 5-FHPA **10** in *Streptomyces* sp. MA37.

A homolog search of the *Streptomyces* sp. MA37 genome revealed an open reading frame (orf) *fdrA* that is predicted to encode a metal-dependent phosphoesterase, sharing high sequence identity (56% identity) with SalN of the salinosporamide biosynthetic pathway. Notably, immediately downstream of *fdrA* are two orfs, *fdrB* and *fdrC*, that are divergently transcribed. These are FdrB and FdrC which also share high sequence identities with SalH (68% identity) and SalM (69% identity), respectively (Fig. 2 and S1†) and are predicted to encode a dihydroxy-acid dehydratase and a short chain dehydrogenase (SDRs). SalM was found to catalyse the NAD^+^ dependant oxidation of 5-ClR **14** to 5-chlororibolactone **15**, which is then hydrolysed to 5-chlororibonate **16** during studies exploring the biosynthesis of salinosporamide A **11**.^21^

**Fig. 3:**
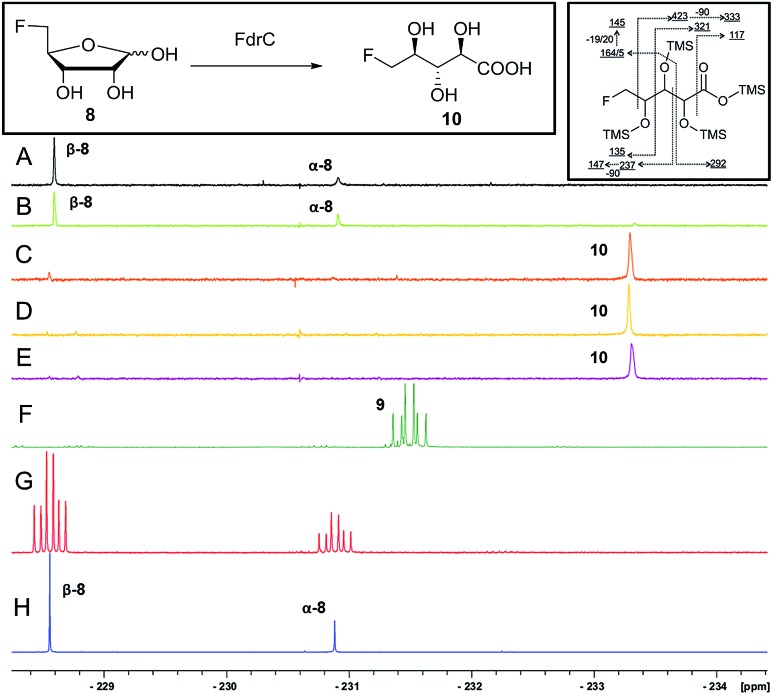
^19^F NMR spectroscopic analyses of (A) ^19^F{^1^H}-NMR of FdrC after overnight incubation with 5-FDR **8** at 37 °C; (B) ^19^F{^1^H}-NMR of the FdrC enzymatic reaction after overnight incubation with NADP^+^ and 5-FDR **8** at 37 °C; (C) ^19^F{^1^H}-NMR of the FdrC enzymatic reaction after overnight incubation with NAD^+^ and 5-FDR **8** at 37 °C; (D) ^19^F{^1^H}-NMR of FdrC enzymatic reaction after overnight incubation with NAD^+^ and 5-FRL **9**; (E) ^19^F{^1^H}-NMR of aqueous solution of **9** after overnight incubation at 37 °C; (F) ^19^F-NMR of synthetic 5-FRL **9**; (G) ^19^F-NMR of synthetic 5-FDR **8**; (H) ^19^F{^1^H}-NMR of synthetic 5-FDR **8**. Left insert: the enzymatic reaction; right insert: the GC-MS fragmentation pattern of 5-FHPA **10**.

**Scheme 3:**
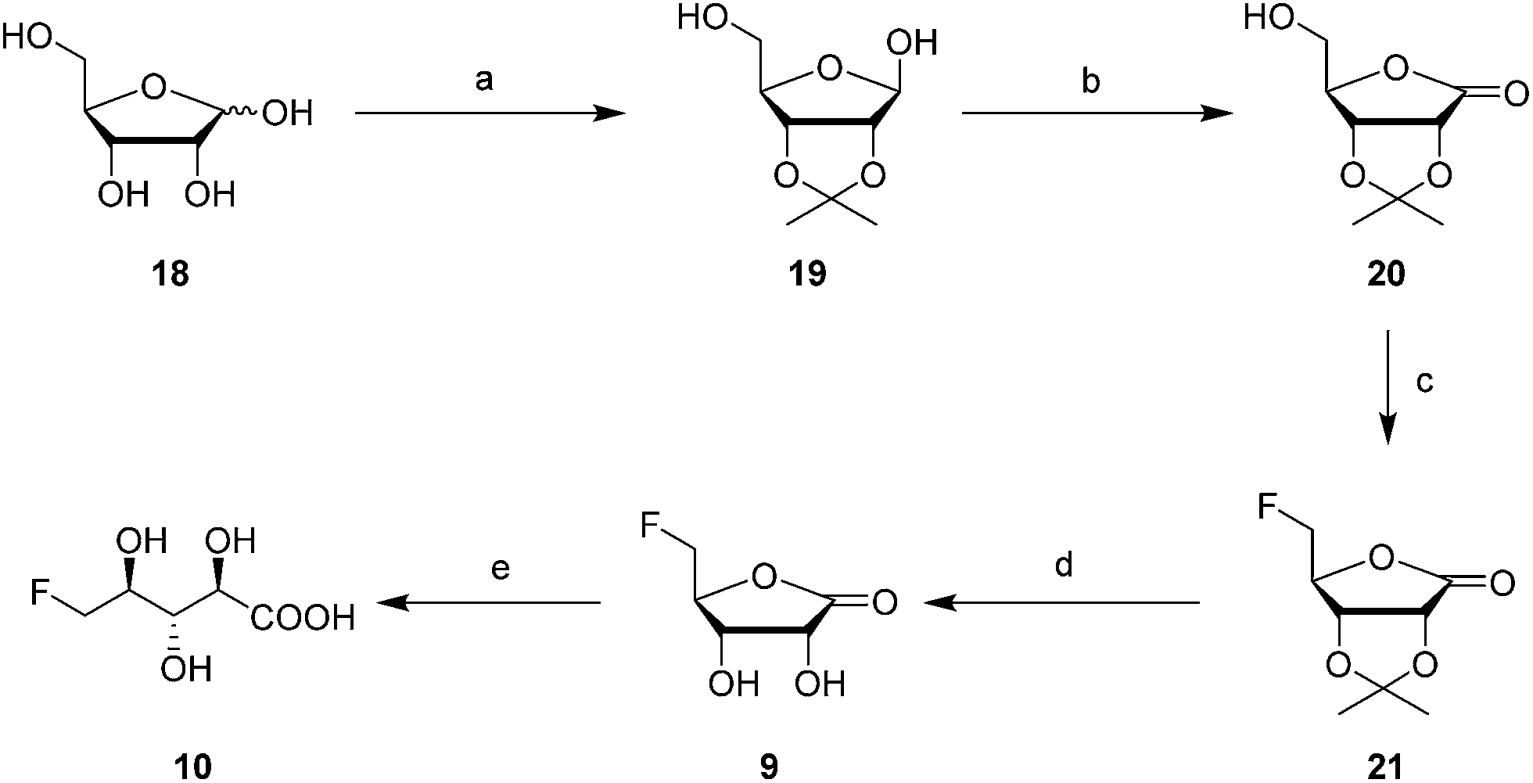
Reagents and conditions: (a) Acetone, conc. H_2_SO_4_ (cat.), 2 h, 24%; (b) I_2_, K_2_CO_3_, DCM, 2 h, 97%; (c) Deoxo-fluor®, DCM, 40 °C, 30 min, 52%; (d) TFA, water, 5 h, 96%; (e) LiOH, water, 3 day, quant.

It was an objective in this study to explore the function of *fdrC*. Over-expression of a codon-optimized synthetic gene for *fdrC* in *E. coli*, with a His_6_ tag and a TEV protease cleavage site added, allowed isolation and purification of the coded protein. The resultant FdrC appeared on SDS-page with an estimated mol. wt ∼31 kDa (Fig. S2†). Incubations of the recombinant enzyme with 5-FDR **8** and NAD^+^ or NADP^+^, were followed by ^19^F NMR spectroscopy. When the assay was conducted in the absence of NAD^+^ or in the presence of NADP^+^, there was no turnover (Fig. 3A and B, respectively). Reactions with NAD^+^ however resulted in an efficient conversion of 5-FDR **8** to a new organofluorine compound (^19^F-NMR; –233.15 ppm, dt, ^2^*J*_HF_ = 25 Hz, ^3^*J*_HF_ = 47 Hz) demonstrating that FdrC is a NAD^+^ dependent enzyme (Fig. 3C). Given the similarity of this pathway to that in salinosporamide-A **11**, it was anticipated that the enzymatic product might be carboxylic acid **10**. This would arise by oxidation of 5-FDR **8** to lactone 5-FRL **9** and then hydrolysis to the ring opened carboxylic acid 5-FHPA **10**. The identity of 5-FHPA **10** in the FdrC reaction mixture was confirmed by comparison with a synthetic sample. A synthesis of 5-FHPA **10** was carried out following the protocol^22^ illustrated in Scheme 3.

The 2′- and 3′-hydroxyl groups of d-ribose **18** were protected by preparation of acetonide **19**.^22^ Oxidation of the anomeric alcohol to give lactone **20** was efficiently accomplished using iodine in DCM in the presence of potassium carbonate,^23^ and then fluorination with Deoxofluor® afforded fluorolactone **21** in good yield.^24^ Acetonide hydrolysis using TFA in water gave 5-FRL **9** and finally hydrolysis was accomplished with aqueous LiOH to give **10**. This synthetic 5-FHPA **10** was found to be identical by ^19^F-NMR to the enzyme (FdrC) reaction product.

**Fig. 4:**
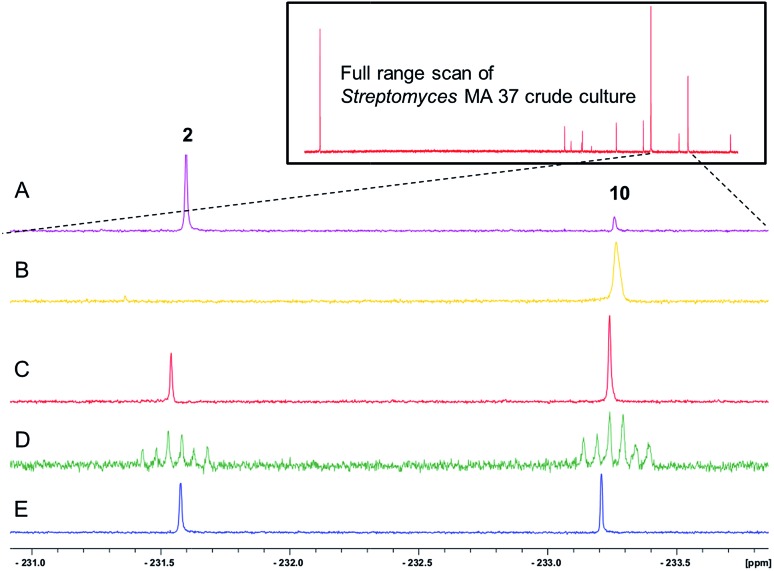
^19^F{^1^H}-NMR spectroscopic analyses of (insert) fluorometabolites in the supernatant of the culture medium from *Streptomyces* sp. MA37; (A) the ^19^F NMR expansion showing two ^19^F NMR signals of 4-FT **2** and 5-FHPA **10**; (B) the FdrC enzymatic reaction after overnight incubation with NAD^+^ and 5-FDR **8**; (C) co-addition of the A and B samples showing the identical chemical shifts; (D) co-addition of synthetic 5-FHPA **10** and sample A with {^1^H} couplings; (E) co-addition of synthetic 5-FHPA **10** and sample A with {^1^H} decoupling.

The ^19^F-NMR signals became coincident when a sample of the enzyme reaction mixture was spiked into the supernatant of the *Streptomyces* sp. MA37 fermentation (Fig. 4C). The synthetic reference of 5-FHPA **10** was also used to confirm this species as a component of the product mixture from *Streptomyces* sp. MA37. This was achieved by global persilylation of the components of the CFE using *N*-methyl-*N*-(trimethylsilyl)-trifluoroacetamide (MSTFA). The extract was then analysed by GC-MS (Fig. 3 and S3†). Comparison with the persilylated derivative of synthetic 5-FHPA **10** revealed a constituent of the CFE with an identical fragmentation pattern and retention time (Fig. S3†). Therefore these studies are consistent with 5-FHPA **10** as the product of FdrC enzyme, and as an end product in fluorometabolite biosynthesis in *Streptomyces* sp. MA37.

Incubation of synthetic 5-FRL **9** with FdrC (Fig. 3D) resulted in its complete conversion to **10**, suggesting lactone hydrolysis is also catalysed by the enzyme. Two step enzymatic catalysis is probably also the case for SalM (conversion of **15** to **16**) on the salinosporamide A **11** pathway.^25^

A study of the reaction kinetics for FdrC mediated NAD^+^ oxidation indicates that FdrC has a higher affinity for d-ribose over 5-FDR **8** as measured by *K*_m_, but overall both riboses oxidise with a similar efficiency (*k*_cat_/*K*_m_) of less than 0.4 μm^–1^ min^–1^ (Table 1 and Fig. S4†).

**Table 1:** Kinetic data of FdrC mediated oxidation using 5-FDR **8** and d-ribose **18** as substrates, respectively

	*V*_max_ (μM min^–1^)	*K*_m_ (μM)	*k*_cat_ (min^–1^)	Specificity constant (*k*_cat_/*K*_m_) (μM^–1^ min^–1^)
5-FDR **8**	4.96 ± 0.25	2.73 ± 0.73	0.58	0.21
d-ribose **18**	2.73 ± 0.05	0.84 ± 0.14	0.32	0.38

**Scheme 4:**
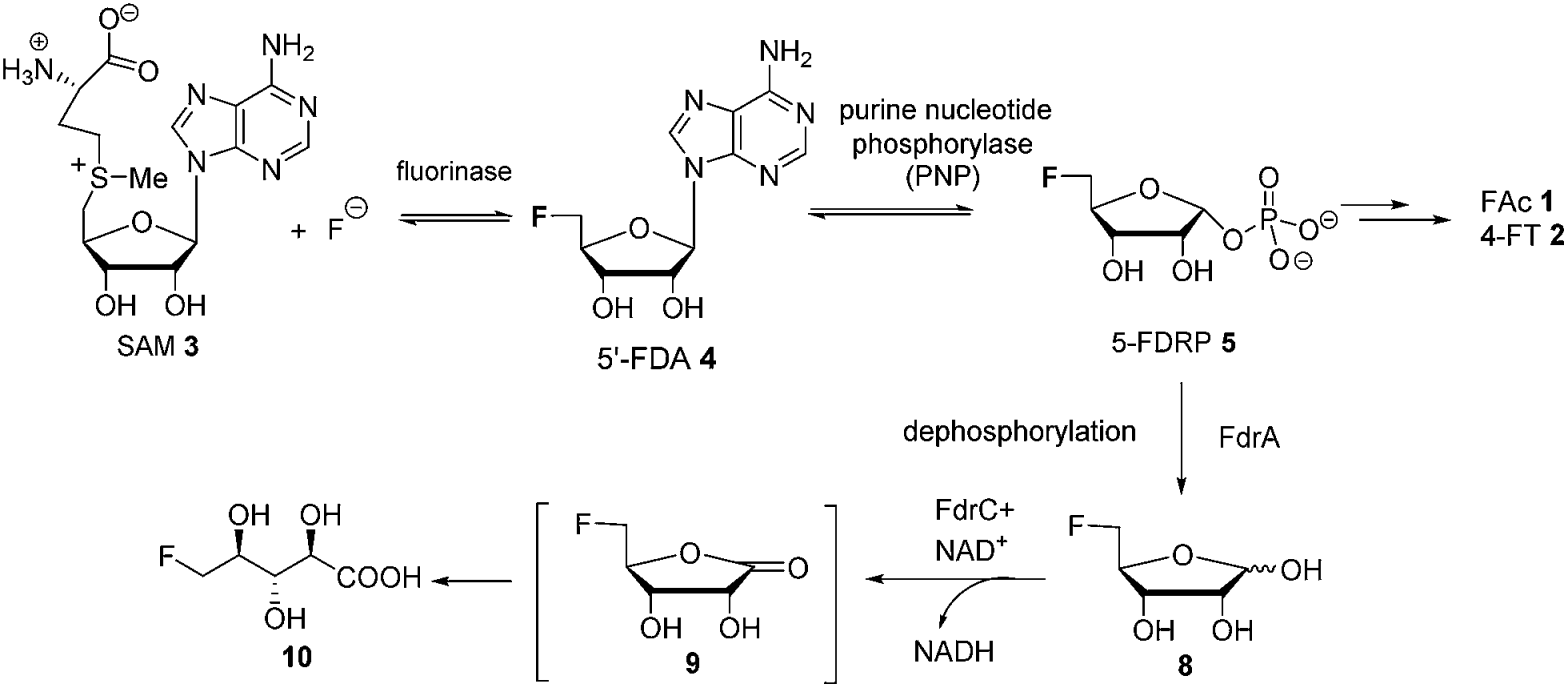
The proposed fluorometabolite pathway toward the synthesis of 5-FHPA **10** from SAM **3** in *Streptomyces* sp. MA37. Bracket: **9**, although synthesized, is an unstable intermediate in the aqueous solution and was not observed in the supernatant of the MA37 fermentation during the ^19^F NMR analysis.

This study identifies **10** as a novel fluorometabolite and extends the very small collection of this rare class of natural products. Organic chemists have only identified five unique fluorine containing natural products so far.3*a* The pathway to **10** branches from that already established for FAc **1** and 4-FT **2** biosynthesis. The branch point occurs at 5-FDRP **5**, whereby phosphorolysis generates 5-FDR **8**. This sugar is then oxidised by FdrC to 5-FRL **9**, which undergoes hydrolysis to generate 5-FHPA **10** (Scheme 4).

We finish with a commentary on the genes that surround *fdrA*–*C* in the gene cluster (Fig. 2). The *fdr* cluster was found located in a different scaffold of the draft genome assembly of the MA37 strain, indicating that it is physically remote to the *fl* gene cluster in the previous report.^13^ The orf *fdrD* is located immediately downstream of *fdrC*, encoding a putative cyclase that shares high sequence identity (61% identity) with SalO. Knockout of *salO* had no obvious effect on salinosporamide A **11** production,^25^ suggesting that *salO* is not involved directly in salinosporamide A synthesis, so a role for FdrD is not obvious. The gene *fdrE* is a LuxR family regulator gene and it shares moderate sequence identity (38% identity) with SalRII, a pathway-specific regulator in the biosynthesis of salinosporamide A. Over-expression of SalRII led to a significant increase in the production of salinosporamide A **11** thus there are perhaps prospects for up-regulation of fluorometabolism by over-expression of this gene.^25^ Immediately upstream of *fdrA* lie two orfs, *fdrF* and *fdrG*. FdrG belongs to the ABC transporter family and an obvious role is not clear. The gene *fdrF* is predicted to encode a ribulose-5-phosphate-4-epimerase, which may be relevant to the downstream metabolism of 5-FDR **8**. Interestingly, the main difference between the newly-identified *fdr* cluster in the MA37 and the *sal* cluster in *S. tropica* is that there is no homolog of *salQ* in the proximity of the *fdr* cluster or anywhere in the draft genome of MA37. SalQ, a putative α-oxoacid ferredoxin oxidoreductase, was proposed to be the key enzyme catalysing oxidative decarboxylation from a 5-carbon intermediate 5-chloro-hydroxy-2-oxopentanoate **17** to a 4-carbon intermediate 4-chloro-3-hydroxybutyryl-CoA **21**. This observation suggested that 5-FHPA **10** cannot be metabolised further and accumulated extracellularly, consistent with our chemical identification of 5-FHPA **10** as one of the most abundant fluorometabolite in the supernatant of the MA37 culture. We are currently focussing on establishing the biochemical steps and structure of the other fluorometabolites on this branch pathway.

## Conclusions

In conclusion, 5-FHPA **10** is identified as a new fluorometabolite branching from the established fluorinase pathway to FAc **1** and 4-FT **2**. *In silico* analysis enabled identification of a biosynthetically relevant gene cluster. *In vitro* assay of over-expressed FdrC demonstrated that it can oxidise (NAD^+^ dependant) 5-FDR **8** to its corresponding 5-FRL **9** followed by hydrolysis to generate 5-FHPA **10**. FdrC was similarly active with d-ribose. GC-MS analysis and correlation of synthetic or enzymatically prepared 5-FHPA **10** with the product of the supernatant of *Streptomyces* sp. MA37 indicated identical products, demonstrating that 5-FHPA **10** is a new natural product of *Streptomyces* sp. MA37. This is the first secure identification of a new fluorinated natural product since 1998.^26^–^29^

## Experimental section

### ^19^F-NMR analysis

The samples from enzyme reaction and the supernatants of *Streptomyces* sp. MA-37 fermentation were subjected to ^19^F-NMR analysis. The ^19^F-NMR spectra were recorded with and without proton decoupling on a Bruker AV-500 MHz instrument (^19^F at 470.3 MHz). The chemical shifts of ^19^F-NMR were calculated with respect to CFCl_3_.

### Cell-free extraction (CFE) reaction

The cells of *Streptomyces* sp. MA 37 was harvested by centrifugation (13 000 rpm × 20 min) after 8 day fermentation. The cell pellets were washed three times using Tris–HCl buffer (20 mM, pH 7.5) supplemented with 10 mM MgCl_2_ to remove remnant culture media. The cells were re-suspended in the same buffer (0.1 g wet-cell weight per mL). The cells were disrupted by ultra-sonication (60% duty cycle for 30–60 s). Cell debris was removed by centrifugation (13 000 rpm, 30 min) and the resultant clear supernatant was used as the cell free extract for incubation experiments. The cell free extracts (1 mL) were supplemented with or without 5-FDR at 37 °C for 6 hours. At the end of the incubation period, protein was precipitated by heating the vial to 90 °C for 3 min and the protein was then removed by centrifugation. The supernatant was collected for ^19^F-NMR analysis.

### Plasmid construction, over-expression and purification of *Streptomyces* sp. MA 37 FdrC enzyme

The plasmid encoding the chemically synthesised and codon-optimised *fdrC* gene was provided by the commercial supplier DNA 2.0 (CA, USA). AGGAGGTAAAACAT was incorporated as the ribosome binding site (RBS). T7 promoter and kanamycin-resistance gene were incorporated. A His-tag containing peptide (Met-Ser-Tyr_2_-His_6_-Asp-Tyr-Asp-IIe-Pro-Thr_2_) was fused to the *N*-terminus of the enzyme. A TEV proteinase cleavage site (Pro-Val-Phe-Ser-Gly) was engineered into the synthetic gene to enable cleavage of the His-tag. Two restriction sites of *Nco*I and *EcoR*I were designed to locate upstream and downstream of the *fdrC* gene, respectively. The plasmid was transformed using the heat shock method. A mixture of chemically competent bacteria and plasmid DNA was placed at 42 °C for 90 s and then placed back on ice. *E. coli* BL21(DE3) Gold cells, transformed with the synthetic *fdrC* plasmids were grown in Lysogeny broth containing 50 mg mL^–1^ kanamycin at 37 °C until cell density reached an absorbance at ∼0.6 at 600 nm. The culture was then cooled on ice for 30 min. A final concentration of 0.3 mM isopropylthiogalactoside (IPTG) was added as induction procedure for FdrC over-expression. The incubation was continued at 25 °C for ∼24 h. The resultant *E. coli* cells were collected and lysed. After centrifugation, the cell lysate was applied onto a bench-top column packed with Ni^2+^-charged His-Bind resin (Qiagen) for protein purification. Recombinant protein bound on the resin was firstly washed with a solution of Tris–HCl (20 mM, pH 8.0), imidazole (20 mM) and NaCl (0.5 M) buffer, followed by a further wash with a solution of Tris–HCl (20 mM, pH 8.0), imidazole (50 mM) and NaCl (0.5 M). The protein was eventually eluted with a solution of Tris–HCl (20 mM, pH 8.0), imidazole (400 mM) and NaCl (0.5 M). The protein concentration was measured by OD_280 nm_ (Nanodrop). The extinction coefficient was determined using the ExPAsy ProtParam tool. The identity of the protein was confirmed by both polyacrylamide gel electrophoresis and ESI-MS analysis (Fig. S2†).

### ESIMS analysis of FdrC enzyme reaction

ESI-FT-MS was used to determine the identity of enzymatic products in the FdrC-mediated reactions. Reaction mixtures consisted of FdrC (0.625 mg mL^–1^), NAD^+^ (5 mM) and MgCl_2_ (10 mM) in the presence of either 5-FDR (10 mM) or d-ribose (10 mM) in the Tris–HCl buffer (1 mL, 25 mM, pH 7.8). The reactions were conducted overnight at 37 °C. Protein was removed by heating the vial to 90 °C for 3 min, followed by centrifugation (133 000 rpm, 3 min). The supernatant was subjected for ESI-FT-MS analysis.

### Enzyme kinetics measurements for FdrC

A UV-Vis spectroscopy-based assay was employed to monitor the enzyme activity of FdrC. The enzyme reactions were initiated by mixing FdrC (final concentration was 0.26 mg mL^–1^) with either 5-FDR or d-ribose, supplemented with 1 mM magnesium chloride and 2.5 mM nicotinamide adenine dinucleotide (NAD^+^) in the Tris–HCl buffer (25 mM, pH 7.8). The concentrations of the substrates used in the assay ranged from 0.5 μM to 200 μM. After mixing with the substrate, the UV spectra were recorded immediately, scanning from 300 nm to 450 nm every 0.2 minutes for 3 min at ambient temperature. An increase in absorbance at 340 nm was attributed to the production of NADH. Initial FdrC reaction velocities were plotted against substrate concentration and a Michaelis–Menten plot was established (see Fig. S4†). Enzyme kinetics parameters *V*_max_, *K*_m_, *k*_cat_, and specificity constant were all calculated accordingly and are listed in Table 1.

## Supplementary Material

Supplementary information
