# Supplementary material for: Identification of a fluorometabolite from Streptomyces sp. MA37: (2R3S4S)-5-fluoro-2,3,4-trihydroxypentanoic acid
Source: Chem Sci. 2014 Dec 3;6(2):1414–9. doi: 10.1039/c4sc03540b (PMC5947533; doi:10.1039/c4sc03540b)
Supplement: Supplementary file 1 [file SC-006-C4SC03540B-s001.pdf]

Electronic Supplementary Material (ESI) for Chemical Science.
This journal is © The Royal Society of Chemistry 2014

Identification of a fluorometabolite from *Streptomyces sp*. MA37: (*2R,3S,4S*)-5-Fluoro-2,3,4-trihydroxypentanoic acid

Long Ma[a], Axel Bartholome[a], Ming Him Tong[b], Zhiwei Qin[b], Yi Yu[c], Thomas Shepherd[d], Kwaku Kyeremeh[e], Hai Deng[b]* and David O'Hagan[a]*

[a]EaStChem School of Chemistry, University of St Andrews, North Haugh, St Andrews KY169ST, United Kingdom.

[b]UK Marine Biodiscovery Centre, Department of Chemistry, Meston Walk, University of Aberdeen, Aberdeen AB24 3UE.

[c]Key Laboratory of Combinatory Biosynthesis and Drug Discovery (Ministry of Education), School of Pharmaceutical Sciences, Wuhan University, 185 East Lake Road, Wuhan 430071 P. R. China.

[d]The James Hutton Institute, Invergowrie, Dundee, DD2 5DA, UK.

[e]Department of Chemistry, FGO Torto Building, University of Ghana, Legon, Ghana.

[ǂ]Currect address: School of Biotechnology, Tianjin University of Science & Technology, Tianjin 300457, P. R. China.

Corresponding authors:

Professor David O'Hagan
School of Chemistry and Centre for Biomolecular Sciences,
University of St Andrews,
North Haugh, St Andrews KY169ST, United Kingdom.
Phone: +44 1334 467176
Email: do1@st-andrews.ac.uk

Dr Hai Deng
Department of Chemistry,
University of Aberdeen,
Aberdeen AB24 3UE,
United Kingdom.
Phone: +44 1224 272953
Email: h.deng@abdn.ac.uk

1. Sequence alignment

Sequence alignment of FdrC (termed Frib/1 in the annotation) and SalM (a short-chain dehydrogenase/reductase enzyme from the marine actinomycete *Salinispora tropica*) was performed using JalView software. This alignment of FdrC showed a 69% similarity to SalM.

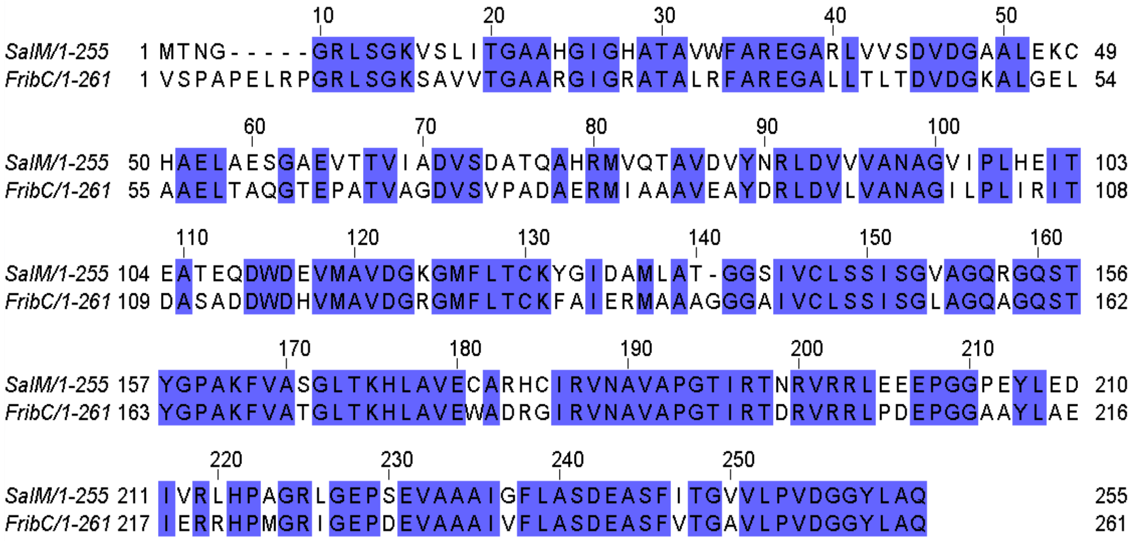

Figure S1. Primary sequence alignment of FdrC (termed FribC/1 in the annotation) with SalM (a short-chain dehydrogenase/reductase enzyme from the marine actinomycete *Salinispora tropica*).

2. MALDI-TOP MS analysis of purified FdrC protein

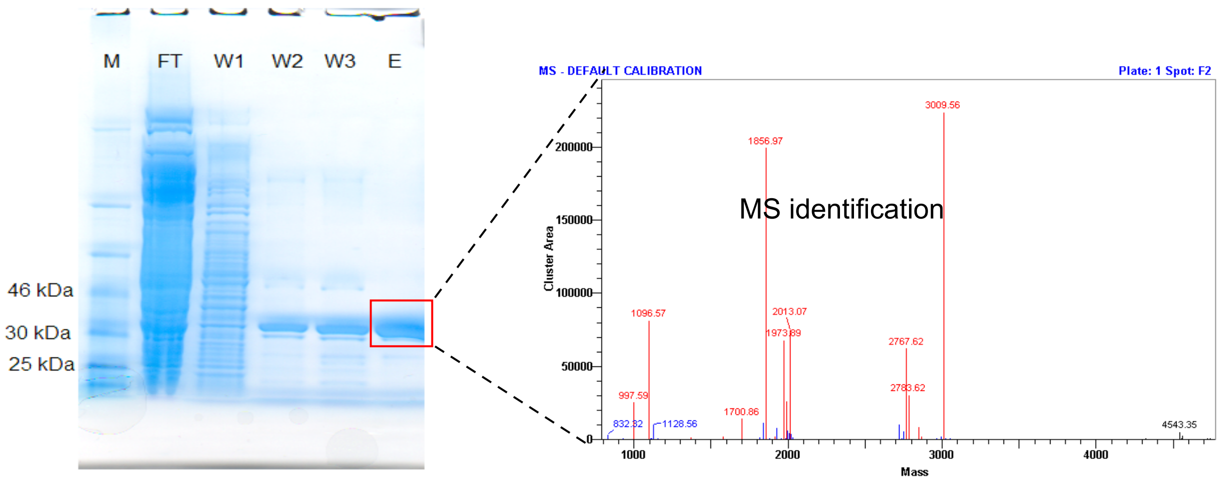

Figure S2. SDS-PAGE of overexpressed FdrC (left), and the ESI-MS identification of purified FdrC (right) (M = molecular marks; FT = flow through; W = wash fraction; E = elution fraction). The gel band was excised and cut into 1mm cubes. These were then subjected to in-gel digestion, using a ProGest Investigator in-gel digestion robot (Genomic Solutions, Ann Arbor, MI) using standard protocols[1]. Briefly the gel cubes were destained by washing with acetonitrile and subjected to reduction and alkylation before digestion with trypsin at 37 °C. The peptides were extracted with 10 % formic acid. The digest solution (0.5 mL) was applied to the MALDI target along with alpha-cyano-4-hydroxycinnamic acid matrix (0.5 mL, 10 mg/mL in 50:50 acetonitrile: 0.1 % TFA) and allowed to dry.

MALDI MS was acquired using a 4800 MALDI TOF/TOF Analyser (ABSciex, Foster City, CA) equipped with a Nd:YAG 355 nm laser and calibrated using a mixture of peptides. The spot was initially analysed in positive MS mode between 800 and 4000 m/z, by averaging 1000 laser spots. The most intense peptides (up to 15) were selected for MSMS analysis and acquired to a maximum of 3000 laser shots or until the accumulated spectrum reached an S/N ratio of 35. All MS/MS data were acquired using 1 keV collision energy.

The MS data were analysed, using GPS Explorer (ABSciex) to interface with the Mascot 2.1 search engine (Matrix Science, London, UK), against the Swiss-Prot and internal databases. No species restriction was applied. The data was searched with tolerances of 100 ppm for the precursor ions, trypsin as the cleavage enzyme, assuming up to one missed cleavage, carbamidomethyl modification of cysteines as a fixed modification and methionine oxidation selected as a variable modification. The MSMS data was also analysed using the Mascot search engine using similar criteria.

3. **GC-MS analysis for the identification of 5-fluoro-2,3,4-trihydroxypentanoic acid (5-FHPA 10) from the supernatant of the *Streptomyces sp.* MA 37 fermentation.**

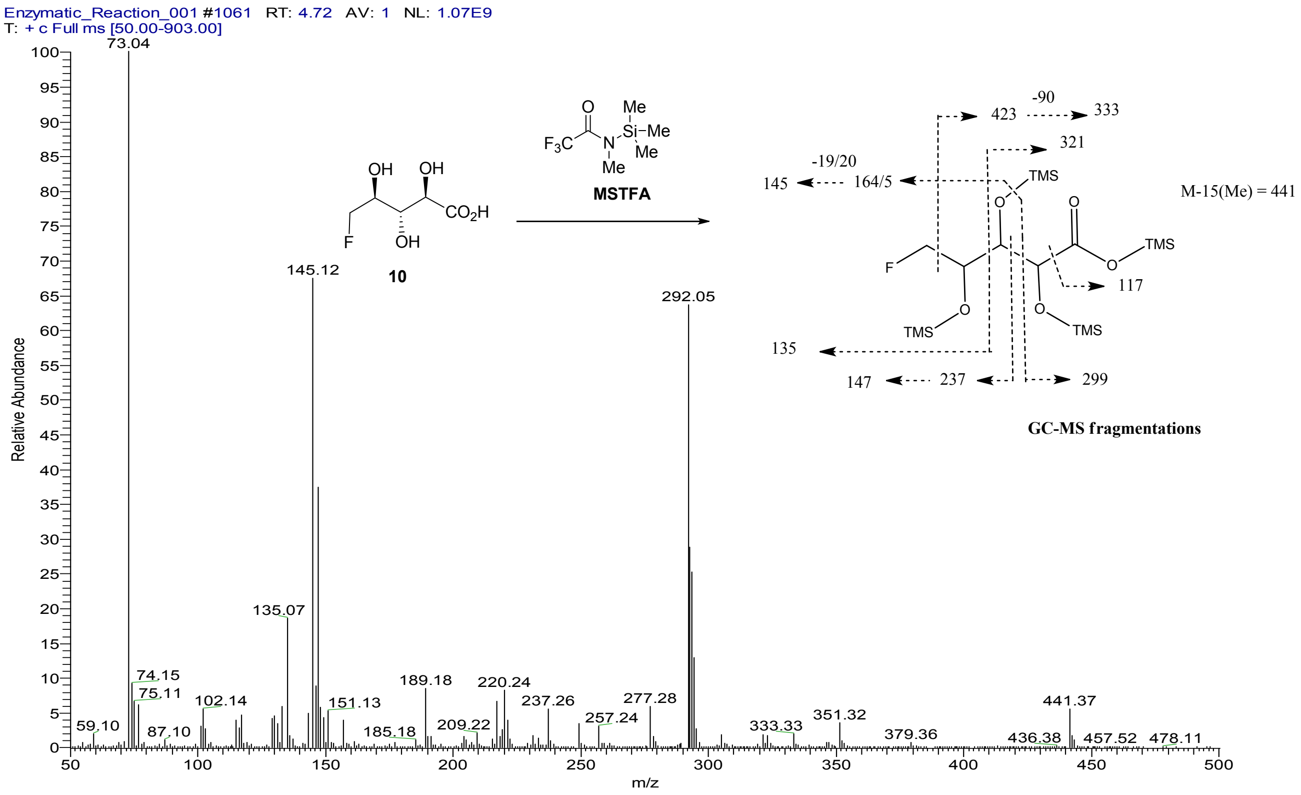

Figure S3. GC-MS fragmentation analysis of MSTFA treated extract shows the mass spectrum and fragmentation pattern of 5-fluoro-2,3,4-trihydroxypentanoic acid $(TMS)_4$.

Freeze dried samples of enzymatic reaction product (5.6 mg), and bacterial culture product (29.8 mg) were transferred to a glass culture tube fitted with a screw cap and PTFE seal. To this 100 µL *N*-methyl-*N*-trimethylsilyltrifluroacetamide (MSTFA, ThermoFisher, UK) and 100 µL of anhydrous pyridine (Sigma-Alrich, UK) was added. The tube was heated on a hot block at 80 ºC for 1 h. The derivatised sample solution was filtered through tissue paper held in the constriction of a Pasteur pipette, which had been pre-washed with dichloromethane, directly into an autosampler vial for analysis by GC-MS. Samples were analysed using a Trace DSQII GC-MS system (Thermo Scientific, Hemel Hempstead, UK) fitted with a CombiPalautosampler (CTC Analytics, Switzerland). Samples (1 µL) were injected into a programmable temperature vaporising (PTV) injector fitted with a Merlin Microseal™ high pressure septum (Agilent Technologies, UK) and operating at a split of 40:1. The PTV conditions were injection temperature 132 ºC for 1 min, transfer rate 14.5 ºC s^{-1}, transfer temperature 320 ºC for 1 min, clean rate 14.5 ºC s^{-1} and clean temperature 400 ºC for 2 min. Chromatography was effected on a DB5-MS™ column (15 m × 0.25 mm × 0.25 µm; Agilent Technologies, UK) using helium at 1.5 mL/min (constant flow). The GC temperatures were 100 ºC for 2.1 min, 25 ºC min^{-1} to 320 ºC then isothermal for 3.5 min. The GC-MS interface temperature was 250 ºC. MS acquisition conditions were electron impact (EI) ionisation at 70 eV, solvent delay 1.25 min, source temperature 200 ºC, mass range 50-900

a.m.u. at 6 scans s^{-1}. Data were acquired using the Xcalibur™ software package V. 2.07 (Thermo Scientific, Hemel Hempstead, UK).

4. Kinetics profiles for FdrC

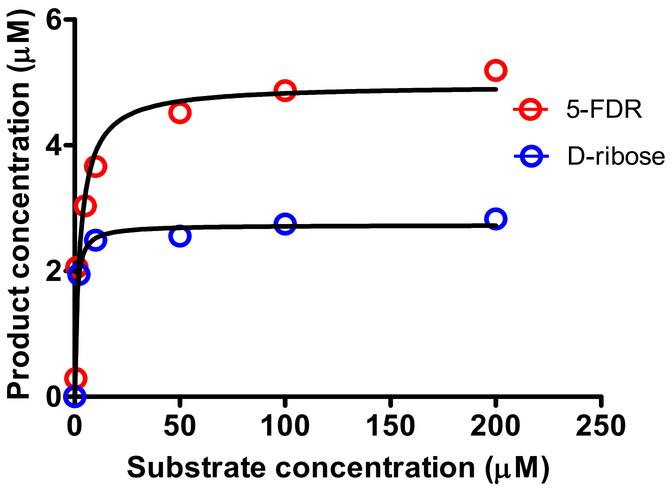

Figure S4. Michaelis-Menten curve of FdrC for 5-fluorodeoxyribose (5-FDR) and D-ribose.

5. Fermentation condition of screening fluorometabolites in *Streptomyces sp* MA37

For metabolite analysis, spores of *Streptomyces sp* MA37 were inoculated into ISP2 liquid medium (1% malt extract, 0.4% yeast extract, 0.4% D-glucose, pH 7.0, 50 mL) in an Erlenmeyer flask (250 mL) and cultivated in a rotary incubator (180 rpm, Barnstead Lab-line MaxQ 5000) at 28 ^{o}C. After 7-day cultivation, the seed culture was then transformed to a production culture in 6 different cultivation media supplemented with 2.5 mM KF. The fermentation media include International *Streptomyces* Protocol (ISP) 2-6 [2] and a defined medium previously reported [3] for the fluorometabolite production in *Streptomyces cattleya*. After a 12-day cultivation, the supernatants were then subjected to $^{19}F\{^{1}H\}$ NMR analysis (Bruker Avance 500 MHz in D_2O).

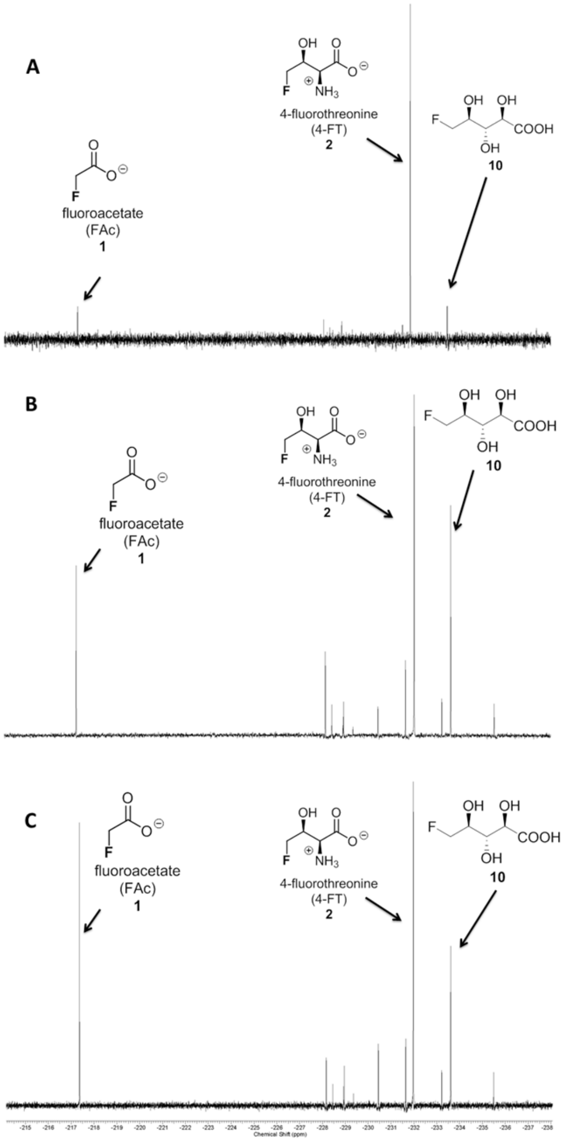

Figure S5 ^{19}F NMR time course analysis of the supernatants of MA37 ISP2 medium supplemented with 2.5 mM KF, indicating that FAc **1**, 4-FT **2** and 5-FHPA **10** are stable fluorometabolites over the cultivation period of up to 19 days. (A) 8-day culture; (B) 12 day culture; (C) 19-day culture.

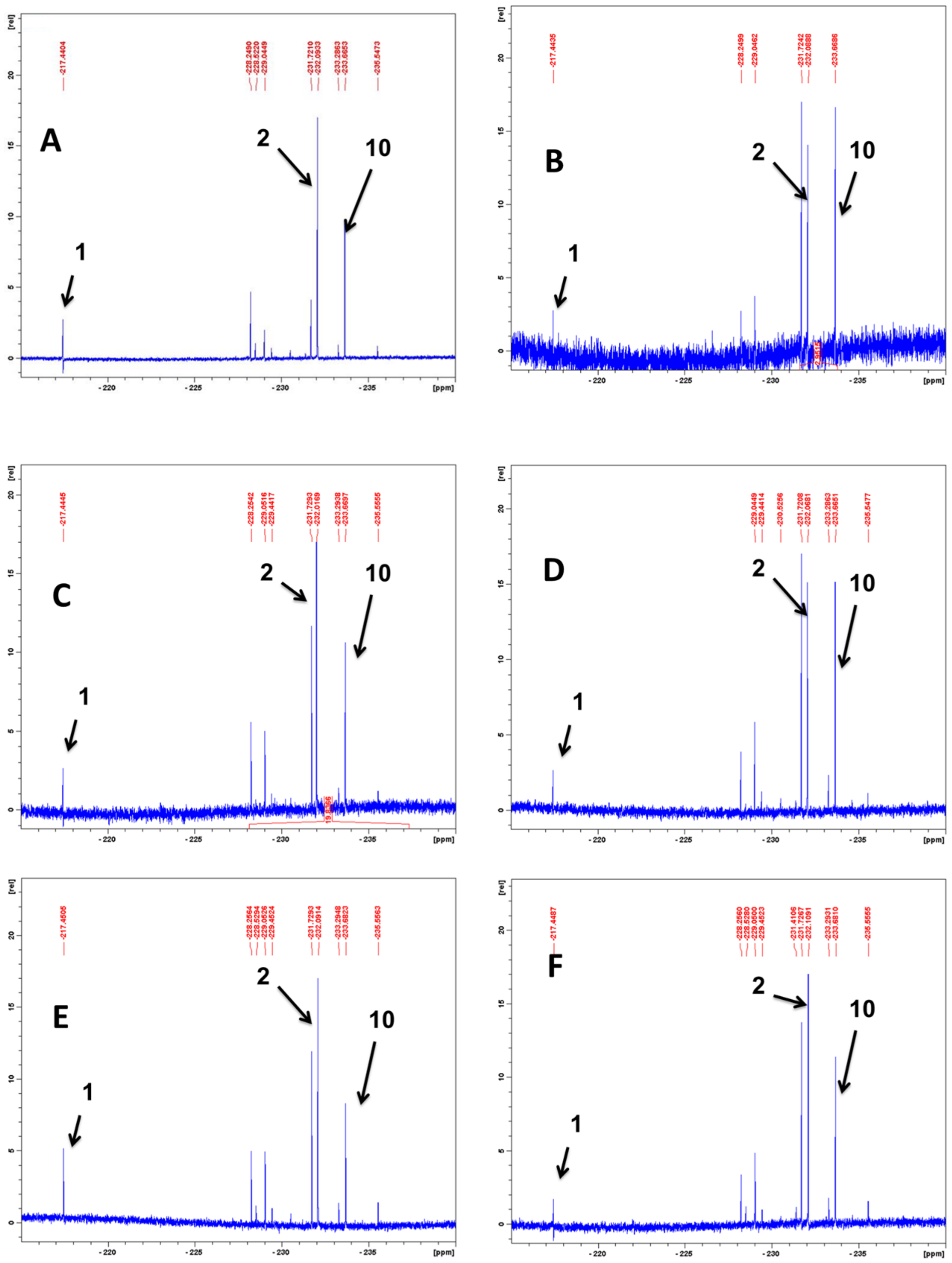

Figure S6 ^{19}F NMR analysis of the supernatants of MA37 culture, indicating that FAc **1**, 4-FT **2** and 5-FHPA **10** are stable fluorometabolites in six cultivation media: (A) ISP 2 media; (B) ISP 3 media; (C) ISP4 media; (D) ISP5 media; (E) ISP6 media; (F) a defined medium previously reported for the fluorometabolite production in *Streptomyces cattleya*.[3]

6. Synthesis of (2*R*,3*S*,4*S*)-5-fluoro-2,3,4-trihydroxypentanoic acid 10

2,3-*O*-Isopropylidene-D-ribose 12

A suspension of D-ribose **11** (3.0 g, 20.0 mmol, 1 eq.) in acetone (30 mL) was cooled to 0 °C before 2,2-dimethoxypropane (2.5 mL, 20.6 mmol, 1.03 eq.) and 4-toluene sulfonic acid (0.19 g, 1.0 mmol, 0.05 eq.) were added. After stirring at room temperature for 1 h, the clear mixture was neutralised with saturated aqueous sodium bicarbonate solution and filtered through pad of a celite. The filtrate was concentrated and the residue was dissolved in EtOAc (60 mL), and then washed with water (15 mL). The aqueous layer was extracted with EtOAc (2 × 30 mL). The combined organic layer was dried ($MgSO_4$), filtered and the filtrate was concentrated. The crude mixture was purified by column chromatography (EtOAc) to afford 2,3-*O*-isopropylidene-D-ribose **12** (0.91 g, 24 %) as a colorless oil: $\boldsymbol{R_f}$ = 0.38 (EtOAc); $[\alpha]^{20}_D$: −29.8° (*c* 0.5, $CHCl_3$); **^{1}H NMR** ($CDCl_3$, 300 MHz) δ_H 5.31 (1H, s, H-1), 4.73 (1H, d, *J* = 5.9 Hz, H-2), 4.47 (1H, d, *J* = 5.9 Hz, H-3), 4.30 (1H, t, *J* = 3.1 Hz, H-4), 3.68-3.55 (2H, m, CH_2), 1.38 (3H, s, CH_3), 1.21 (3H, s, CH_3); **^{13}C NMR** ($CDCl_3$, 100 MHz) δ_C 112.22 ($\underline{C}(CH_3)_2$), 103.12 (CH, C-1), 87.95 (CH, C-4), 87.03 (CH, C-3), 81.84 (CH, C-2), 63.79 (CH_2, C-5), 26.50 (CH_3), 24.85 (CH_3); **IR** ν_{max} (neat): 3419, 2987, 2942, 1458, 1377, 1212, 1067, 870; **HRMS** m/z (ES^+) (calculated $C_8H_{14}NaO_5^+$ = 213.0733) found 213.0728 $[M+Na]^+$.

2,3-*O*-Isopropylidene-D-ribono-1,4-lactone 13

An aliquot K_2CO_3 (1.99 g, 14.4 mmol, 3 eq.) and iodine (3.65 g, 14.4 mmol, 3 eq.) were added to a solution of 2,3-*O*-isopropylidene-D-ribose **12** (0.91 g, 4.8 mmol, 1 eq.) in CH_2Cl_2 (15 mL). The reaction mixture was stirred at room temperature for 4 h then quenched by addition of a saturated aqueous solution of Na_2SO_3 (10 mL) and the reaction was vigorously stirred for a further 30 min. The organic phase was collected and the aqueous phase extracted into EtOAc (3 × 20 mL). The combined organic extracts were dried ($MgSO_4$) and evaporated. The product was then purified by chromatography (EtOAc) to afford 2,3-*O*-isopropylidene-D-ribono-1,4-lactone **13** (0.88 g, 97 %) as a white solid: $\boldsymbol{R_f}$ = 0.38 (EtOAc); $[\alpha]^{20}_D$: −62.8° (*c* 0.25, $CHCl_3$); **m.p.** 129-133 °C; **^{1}H NMR** ($CDCl_3$, 300 MHz) δ_H 4.84 (1H, d, *J* = 5.6 Hz, H-2), 4.78 (1H, d, *J* = 5.6 Hz, H-3), 4.63 (1H, br s, H-4), 4.00 (1H, d, *J* = 11.9 Hz, H-5), 3.82 (1H, d, *J* = 11.9 Hz, H-5), 1.48 (3H, s, CH_3), 1.39 (3H, s, CH_3); **^{13}C NMR** ($CDCl_3$, 100 MHz) δ_C 175.46 (C=O), 113.34 ($\underline{C}(CH_3)_2$), 82.55 (CH, C-4), 78.36 (CH, C-3), 75.75 (CH, C-2), 62.24 (CH_2, C-5), 26.87 (CH_3), 25.63 (CH_3); **IR** ν_{max} (neat): 3460, 1763, 1377, 1221, 1196, 1153, 968, 854, 808, 773; **HRMS** m/z (ES^+) (calculated $C_8H_{12}NaO_5^+$ = 211.0577) found 211.0572 $[M+Na]^+$.

5-Deoxy-5-fluoro-2,3-*O*-isopropylidene-D-ribono-1,4-lactone 14

Deoxo-fluor ® (50% in THF, 0.62 mL, 1.67 mmol, 3.04 eq.) was slowly added to a stirred solution of 2,3-*O*-isopropylidene-D-ribono-1,4-lactone **13** (0.104 g, 0.55 mmol, 1 eq.) in CH_2Cl_2 (1.5 mL) at room temperature. After stirring at 40 °C for 30 min, ice water was carefully added to quench the excess of Deoxo-fluor ®. The aqueous phase was extracted into EtOAc (3 × 5 mL) and the organic phases combined and dried ($MgSO_4$). The product was then purified by column chromatography (Petrol/EtOAc 3:1) to afford 5-deoxy-5-fluoro-2,3-*O*-isopropylidene-D-ribono-1,4-lactone **14** (55 mg, 52 %) as pale white solid: R_f = 0.55 (EtOAc/PE 1:1); $[\alpha]^{20}_D$: −74.4° (*c* 0.25, $CHCl_3$); **m.p.** 65-68 °C; **^{1}H NMR** ($CDCl_3$, 300 MHz) δ_H 4.82 (1H, d, J = 5.4 Hz, H-2), 4.79 (1H, dd, J = 5.6 Hz, 3.3 Hz, H-3), 4.75-4.59 (3H, m, H-4 & CH_2), 1.41 (3H, s, CH_3), 1.41 (3H, s, CH_3); **^{13}C NMR** ($CDCl_3$, 100 MHz) δ_C 169.90 (C=O), 115.40 ($\underline{C}(CH_3)_2$), 82.58 (CH_2, d, J = 172.0 Hz, C-5), 80.42 (CH, d, J = 18.2 Hz, C-4), 77.37 (CH, d, J = 4.9 Hz, C-3), 75.28 (CH, d, J = 3.8 Hz, C-2), 26.90 (CH_3), 25.75 (CH_3); **^{19}F NMR** ($CDCl_3$, 376 MHz) δ_F -235.50 (1F, dddd, J = 48.5 Hz, 45.3 Hz, 34.4 Hz, 3.3 Hz); **IR** ν_{max} (neat): 3545, 2995, 1788, 1383, 1269, 1055, 847; **HRMS** m/z (ES^+) (calculated $C_8H_{11}FNaO_4^+$ = 213.0534) found 213.0526 $[M+Na]^+$.

5-Deoxy-5-fluoro-D-ribono-1,4-lactone 9

A solution of 5-deoxy-5-fluoro-2,3-*O*-isopropylidene-D-ribono-1,4-lactone **14** (120 mg, 0.63 mmol, 1eq.) in a mixture of TFA/water (5 mL, 9:1) was stirred at room temperature for 5 h. The reaction mixture was co-evaporated five times with Et_2O (5 mL), affording 5-deoxy-5-fluoro-D-ribono-1,4-lactone **9** (90.5 mg, 96 %) as white amorphous solid: $[\alpha]^{20}_D$: +7.6° (*c* 0.25, H_2O); **m.p.** 81-85 °C; **^{1}H NMR**($CDCl_3$, 300 MHz) δ_H4.63-4.60 (1H, m), 4.58-4.48 (3H,m), 4.38 (1H,d, J= 5.5 Hz, H-2); **^{13}C NMR**($CDCl_3$, 100 MHz)δ_C 177.96 (C=O), 84.20 (CH, d, J= 17.8 Hz, C-4), 82.20 (CH_2, d, J= 168.5 Hz, C-5), 68.89 (CH, d, J= 2.1 Hz, C-2), 68.64 (CH, d, J= 5.1 Hz, C-3);**^{19}F NMR** ($CDCl_3$, 376 MHz) δ_F -231.64 (1F, td, J= 46.7 Hz, 33.2 Hz); **IR** ν_{max} (neat): 3429, 3280, 1751, 1190, 1140, 966, 895, 781; **HRMS** m/z (ES^+) (calculated $C_5H_7FO_4$ = 150.0328) found 173.0219 $[M+Na]^+$.

(2*R*,3*S*,4*S*)-5-Fluoro-2,3,4-trihydroxypentanoic acid 10

LiOH (27.8 mg, 0.66 mmol, 1.1 eq.) was added to a solution of 5-deoxy-5-fluoro-D-ribono-1,4-lactone **9** (90.5 mg, 0.6 mmol, 1 eq.) in water (3 mL). The resultant mixture was stirred at room temperature for 72 h. The pH was adjusted to pH=7 by careful addition of dil HCl. The reaction mixture was evaporated, affording (2*R*,3*S*,4*S*)-5-fluoro-2,3,4-trihydroxypentanoic acid **10** as white solid in quantative yield: $[\alpha]^{20}_D$: -4.0° (*c* 0.25, H_2O); **m.p.** 165-168 °C; **^{1}H NMR**($CDCl_3$, 300 MHz) δ_H4.61-4.41 (2H, m, CH_2), 4.04 (1H,d, J= 3.1 Hz, H-2), 3.94-3.82 (2H,m, H-3 & H-4); **^{13}C NMR**($CDCl_3$, 100 MHz) δ_C 177.93 (C=O), 84.97 (CH_2, d, J= 164.6 Hz, C-5), 73.36 (CH, C-2), 72.15 (CH, d, J= 7.3 Hz, C-3), 69.80 (CH, d, J= 17.8 Hz, C-4);**^{19}F NMR**($CDCl_3$, 376 MHz) δ_F -233.46 (1F, td, J= 47.4 Hz, 24.5 Hz); **IR** ν_{max} (neat): 3209, 1604, 1408, 1336, 1096, 1036, 949, 910, 810; **HRMS** m/z (ES^-) (calculated $C_5H_9FO_5$ = 168.0434) found 167.0358 $[M-H]^-$.

References:

1. A. Shevchenko, M. Wilm, O. Vorm, M. Mann, *Anal. Chem.*, 1996, **68**, 850 – 858
2. E.B. Shirling, D. Gottlieb, *Int. J. Syst. Bacteriol*. 1966, **16**, 313-340.
3. K. A. Reid, J. T. G. Hamilton, R. D. Bowden, D. O'Hagan, L. Dasaradhi, M. R. Amin and D. B. Harper, *Microbiology*, 1995, **141**, 1385–1393.
